# Exposure misclassification of self-reported vegetarian diet and its impact on health outcomes

**DOI:** 10.1093/ije/dyag168

**Published:** 2026-10-06

**Authors:** Narges Ghoreishi, Tobias Kurth, Sven Knüppel, Matthew P Fox

**Affiliations:** Institute of Public Health, Charité—University Medical Center Berlin, Berlin, Germany; Department of Exposure, German Federal Institute for Risk Assessment (BfR), Berlin, Germany; Institute of Public Health, Charité—University Medical Center Berlin, Berlin, Germany; Department of Food and Feed Safety in the Food Chain, German Federal Institute for Risk Assessment (BfR), Berlin, Germany; Department of Epidemiology, Boston University School of Public Health, Boston, MA, United States; Department of Global Health, Boston University School of Public Health, Boston, MA, United States

**Keywords:** quantitative bias analysis, differential exposure misclassification, dietary recalls, vegetarian diet, self-report, sensitivity and specificity, positive predictive value, negative predictive value

## Abstract

**Background:**

Literature around the health impact of a vegetarian diet is expanding, yet exposure measurement and its effect on the study results receive far less attention. The aim of our study was to show the effect of bias from exposure misclassification on the direction, amount, and uncertainty of study results.

**Methods:**

We included 11 180 participants from the National Health and Nutrition Examination Survey (2007–10). The exposure was self-reported diet type (being vegetarian). Using sensitivity, specificity, and positive and negative predictive values as bias parameters, we quantified the effect of exposure misclassification in non-differential and differential scenarios. We compared conventional analysis with bias-adjusted results.

**Results:**

Among self-reported vegetarians (2.2%), more than half (68%) reported consuming meat, poultry, or fish in the 24-hour dietary recalls. The sensitivity of self-reported diet type was 32% (95% CI, 0.26–0.38) and the specificity was 98% (95% CI, 0.98–0.99). The exposure misclassification was differential. The quantitative bias analysis of differential exposure misclassification was more informative. Using positive and negative predictive values as bias parameters was helpful to avoid negative bias-adjusted cell counts.

**Conclusion:**

Assessing the impact of exposure misclassification in the self-reported vegetarian diet on four health outcomes, our study demonstrates that quantitative bias analysis can help to make more plausible assumptions about the possible direction, strength, and uncertainty of bias and its impact on the effect estimates of conventional analysis. However, comparing different scenarios showed that the model assumptions play a key role in the informative value of quantitative bias analysis.

Key Messages

- We quantified the exposure misclassification of self-reported diet type (vegetarian versus non-vegetarian) and its impact on four health outcomes by using three probabilistic bias analysis scenarios.
- The exposure misclassification was differential, which notably impacted the bias-adjusted results and increased the uncertainty.
- Our study shows that the effect of exposure misclassification is specific to each misclassification scenario and outcome, and should be quantified for each effect estimate.

## Introduction

Diet is a complex behavior that can affect health [1, 2]. Research on the health effects of a vegetarian diet has grown [3–13]. However, while outcome measurement is more rigorously assessed, exposure measurement error, such as the misclassification of self-reported vegetarians, receives less attention [14]. An individual’s definition of a vegetarian diet may differ from a standardized definition used in a research study, leading to misclassification [15]. An alternative approach is to use repeated 24-hour dietary recall that does not rely on long-term memory and provides open-ended data on dietary intake [16], but only captures a limited number of days.

Misclassification associated with dietary assessments can bias study results, making it critical to quantify the impact of this measurement error. Although most systematic reviews in nutritional epidemiology (60%) consider bias due to exposure misclassification in their interpretation of findings, very few (13%) explicitly discuss the direction or likely magnitude of the bias [17], leaving readers to guess the likely impact.

Quantitative bias analysis (QBA) is a set of methods that can quantify the direction, magnitude, and uncertainty in study results due to systematic error [18]. Although these methods have been around for decades, they are still not routinely used in epidemiological research. In a review of the application of these methods in epidemiological studies, nutritional epidemiology had one of the lowest application rates [19].

To address this limitation, we aim to show the importance of implementing bias analysis for exposure misclassification when studying the effect of diet type (vegetarian versus non-vegetarian) on bone mineral density, blood total cholesterol, depression, and bowel health in the publicly available National Health and Nutrition Examination Survey (NHANES) [20].

## Methods

### Population

We used cycles 2007–08 and 2009–10 of the NHANES, which consists of demographic data, health indicators, and other related information collected during household interviews, health surveys, and physical examinations [20]. We included adults >20 years of age who answered the self-perceived vegetarian question and completed at least one 24-hour dietary recall (24h-DR).

### Exposure assessment

We obtained data on two different measures of the exposure: self-report as a vegetarian (asked in the 2007 and 2009 cycles) and two 24h-DRs asking to recall everything eaten and drunk on the previous calendar day [21]. We defined a vegetarian diet as containing no meat, poultry, or seafood.

We used the 24h-DR as a “reference method” for the validation of a self-reported vegetarian diet. Though not a “gold standard” due to its inherent limitations as a short-term measurement instrument, it is valuable for modeling the potential misclassification error associated with self-reported vegetarian diets where no gold standard exists.

### Exposure classification groups

We defined three different dichotomous exposure classification groups (Table 1) comparing vegetarian to non-vegetarian diet types:

**Table 1:** Classification of exposure in the three exposure groups of self-report, 24-hour dietary recall, and a combination of the two.

Group 1 (self-report)	Group 2 (reference)	Group 3 (combination)
Vegetarian	No meat, poultry, or seafood	Vegetarian
Vegetarian	Meat, poultry, or seafood	Non-vegetarian
Non-vegetarian	No meat, poultry, or seafood	Non-vegetarian
Non-vegetarian	Meat, poultry, or seafood	Non-vegetarian

- Exposure classification Group 1 (self-report): “Vegetarian” is defined as those who self-reported as vegetarian and “non-vegetarian” is defined as those who self-reported as non-vegetarian.
- Exposure classification Group 2 (reference): “Vegetarian” is defined as those who listed no meat, poultry, or seafood consumption in the 24h-DRs. “Non-vegetarian” is defined as those who listed meat, poultry, or seafood consumption in the 24h-DRs.
- Exposure classification Group 3 (combination): “Vegetarian” is defined as those who both self-reported as vegetarian and listed no meat, poultry, or seafood consumption in the 24h-DRs. “Non-vegetarian” is defined as those who self-reported as non-vegetarian and those who self-reported as vegetarian but listed meat, poultry, or seafood consumption in the 24h-DRs. Note that, in this classification, two 24h-DRs do not suffice to label the diet of a person who reported being non-vegetarian as vegetarian.

Exposure classification Groups 1 and 2 represent a single measure of exposure. Group 1 acts as the biased exposure and Group 2 as the reference. Group 3 shows a situation in which the exposure definition is expected to be more specific, as two methods are required to be positive to assign vegetarian status.

### Outcome assessment

We investigated the impact of a vegetarian diet on the four outcomes: (i) bone mineral density, (ii) blood total cholesterol, (iii) depression, and (iv) bowel health. The outcomes were selected based on the reported effect of a vegetarian diet on them [3, 4, 6–13]. Two outcomes were collected via self-report (depression and bowel health), one outcome by physical examination (bone mineral density), and one outcome by using a laboratory test (total blood cholesterol). The methods to measure the outcomes are described in Supplementary section 1.1.

### Statistical analysis

The outcome (diseased versus non-diseased) and exposure status (vegetarian versus non-vegetarian) are modeled as binary variables. We performed weighted logistic regression for each outcome by using the adjustment sets described in the Supplementary material. We used the mobile examination center weights recommended by the National Center for Health Statistics across all models to account for unequal probabilities of selection resulting from the complex survey design and nonresponse [22].

The adjustment variables for every outcome and our approach to missing values are described in Supplementary sections 1.2–1.3.

We used R, version 4.4.1 [23] and R package survey [24] for the weighted logistic regression employing NHANES sampling weights. Sample code for differential and non-differential bias analysis is available in the Supplementary material.

### Quantitative bias analysis

We quantified the impact of measurement error on the magnitude and direction of bias due to exposure (diet type) misclassification and estimated the amount of uncertainty in the estimate of effect associated with misclassification.

We used record-level probabilistic bias analysis (PBA), which allowed us to adjust for misclassification while also adjusting for confounding and using the survey weights in the models. This approach requires specifying prior distributions for the bias parameters of exposure classification. As a result, we defined three PBA scenarios to adjust for information bias from exposure misclassification:

- Scenario 1 (non-differential PBA): assuming non-differential misclassification using sensitivity and specificity as bias parameters.
- Scenario 2 (differential PBA, SE/SP): assuming differential misclassification using sensitivity and specificity as bias parameters.
- Scenario 3 (differential PBA, PPV/NPV): assuming differential misclassification using positive and negative predictive values as bias parameters.

In these three scenarios, the biased exposure of Group 1 classification (self-report) is chosen as the exposure and the Group 2 classification (24h-DRs) as the reference for bias parameters (detailed below). The steps toward applying these three scenarios are described in Table 2. The methods have been previously described in more detail by Fox *et al.* [18]. We used the code provided by Fox *et al.* [25] as a template.

**Table 2:** Steps of PBA using record-level data and sensitivity, specificity, positive predictive value, and negative predictive value.

PBA with SE and SP as bias parameters	PBA with PPV and NPV as bias parameters
(i) Summarize the self-reported exposure into 2 × 2 tables for each exposure–outcome pair and perform simple bias analysis. Simple bias analysis is adjusting the summary-level estimate once with the bias parameters (sensitivity and specificity). In Table 3, results of the simple bias analysis of the exposure misclassification are shown under “reference” (crude odds ratio)	
(ii) Specify probability distributions (we used beta distributions) that describe uncertainty in the values of sensitivity and specificity of self-reported exposure classification (from 24-hour dietary recalls)	(ii) Specify probability distributions (we used beta distributions) that describe uncertainty in the PPV and NPV of self-reported exposure classification (from 24-hour dietary recalls)
(iii) Randomly sample values of sensitivity and specificity from the specified distributions	(iii) Randomly sample from positive and negative predictive values from the specified distributions
(iv) Sample prevalence of exposure using beta distributions parameterized by adjusted data (α = # of exposed and β = # of unexposed)	(iv) Calculate prevalence of exposure using the sampled positive and negative predictive values
(v) Use the sampled values of sensitivity (SE) and specificity (SP) and prevalence to calculate positive and negative predictive values (PPV and NPV) for those with and without the outcome in Scenario 2: $\mathrm{PPV}=Pr(E=1|E^{*}=1,D=d)$ and $\mathrm{NPV}=Pr(E=0|E^{*}=0,D=d)$ Simulate a bias-adjusted exposure variable in each record in the dataset by applying the relevant sampled values of PPV and NPV to each individual’s classified exposure using Bernoulli trials	(v) Simulate a bias-adjusted exposure variable in each record in the dataset by applying the relevant sampled values of PPV and NPV to each individual’s classified exposure using Bernoulli trials
(vi) Fit a multivariable logistic regression to the bias-adjusted data to estimate the bias-adjusted odds ratio and its standard error	
(vii) Add random error to the estimated odds ratio by multiplying a random draw from a standard normal distribution (mean = 0, standard deviation = 1) by the standard error of the log odds ratio and exponentiate the result to obtain the total bias-adjusted odds ratio	
(viii) Save the bias-adjusted odds ratios and repeat 100 000 times to obtain a distribution of bias-adjusted results	
(ix) Summarize the resulting distribution of bias-adjusted results using the distribution median as the point estimate and a 95% simulation interval (95% SI) with the limits as the 2.5th to 97.5th percentiles of the bias-adjusted distribution to show the impact of bias on uncertainty	

NPV, negative predictive value; PPV, positive predictive value; SE, sensitivity; SP, specificity.

### Bias parameters

The bias parameters for Scenarios 1 and 2 were sensitivity and specificity, and those for Scenario 3 were positive predictive value and negative predictive value.

We defined sensitivity (SE) as the fraction of participants who self-reported being a vegetarian among those who reported no meat, poultry, or seafood consumption in 24h-DRs. Thus, if we take the true exposure as E and the misclassified exposure as $E^{*}$, then the sensitivity can be expressed as $Pr(E^{*}=1|E=1)$. Specificity (SP) is the fraction of people who stated that they were non-vegetarians among those who reported meat, poultry, or seafood consumption in 24h-DRs, which can be expressed as $Pr(E^{*}=0|E=0)$.

Consequently, the positive predictive value is the fraction of participants who reported no consumption of meat, poultry, or seafood among self-reported vegetarians and the negative predictive value is the fraction of participants who reported consumption of meat, poultry, or seafood among the self-reported non-vegetarians.

As we do not know the bias parameters with certainty, for PBA, we specify distributions around either SE and SP (Scenarios 1 and 2) or PPV and NPV (Scenario 3) of the exposure classification and sample values from them. We used beta distributions, which are probability distributions defined on the (0, 1) interval with two parameters: α and β. The beta distribution for sensitivity (specificity) is defined as the # true test positives (negatives) as α and the # test negatives (positives) among the true positives (negatives) as β. The beta distribution for positive predictive value (negative predictive value) is defined as the # true test positives (negatives) as α and the # test positives (negatives) among the true negatives (positives) as β.

The α and β parameters for the beta distribution were based on non-differential assumption in Scenario 1 and differential assumption in Scenarios 2 and 3. Non-differential and differential parameters were taken from 2 × 2 tables of self-reported exposure measurement stratified by each outcome. The parameters chosen for each beta distribution are shown in Supplementary Table 1. For Scenario 3, we applied the conventional approach and a second approach using uniform prior beta (α = 1, β = 1) [26].

To ensure the stability and accuracy of our analysis, avoiding the occurrence of negative bias-adjusted cell counts, we have implemented and compared different methods, which are described in Supplementary section 1.4. Scenario 3 does not require such measures because it inherently avoids negative cell counts.

## Results

From the two NHANES cycles 2007–08 and 2009–10, 41% (8533) were <20 years of age, 0.01% (3) had missing self-reported diet type, and 5% (970) were missing both of the 24h-DRs. Of the included 11 180 participants, 2.2% were self-reported vegetarians. The sensitivity and specificity of the self-reported diet type were 32% (95% confidence interval (CI) 26%–38%) and 98% (95% CI 98%–99%), respectively. Eligibility criteria and the number of analytical samples for each outcome are shown in Supplementary Figure 1. Table 3 shows the crude odds ratios of exposure classification Groups 1–3. The result for Group 2 is equivalent to simple bias analysis.

**Table 3:** Two by two tables of exposure classification Groups 1–3 presenting the conventional crude odds ratio for each outcome

Exposure classification	Group 1 (“self-report”)		Group 2 (“reference”)		Group 3 (“combination”)	
Categorized as	Vegetarian	Non-vegetarian	Vegetarian	Non-vegetarian	Vegetarian	Non-vegetarian
**Bone mineral density**						
Osteopenia	62	2277	64	2275	21	2318
Normal	115	5632	113	5634	41	5706
Crude odds ratio (95% CI)	1.34 (0.97, 1.82)		1.40 (1.02, 1.91)		1.27 (0.73, 2.13)	
**Blood total cholesterol**						
High	94	4492	101	4485	24	4562
Normal	140	5760	148	5752	59	5841
Crude odds ratio (95% CI)	0.86 (0.66, 1.12)		0.88 (0.68, 1.13)		0.52 (0.32, 0.83)	
**Depression**						
Depressed	22	911	29	904	4	929
Not depressed	194	8577	190	8581	69	8702
Crude odds ratio (95% CI)	1.07 (0.67, 1.64)		1.45 (0.96, 2.13)		0.56 (0.17, 1.37)	
**Bowel health**						
Not healthy	56	2262	55	2263	12	2306
Healthy	164	7522	169	7517	62	7624
Crude odds ratio (95% CI)	1.14 (0.83, 1.54)		1.08 (0.79, 1.46)		0.65 (0.33, 1.16)	

Among participants classified as vegetarian by self-report, more than half (68%) reported the consumption of meat, poultry, or fish in the 24h-DRs, with the majority (52%) reporting it on both recalls. Among self-reported non-vegetarians, 1.6% reported no consumption of meat, poultry, and fish in the 24h-DRs. The meat/poultry/fish consumption among vegetarians was mostly recorded in the second dietary recall (Supplementary Table 2). Participants either missed the second or both 24h-DRs (Supplementary Table 3). The self-reported exposure had a differential misclassification in all four outcomes.

Compared with vegetarians, more non-vegetarians were male (49% versus 38%) and white (48% versus 40%). They had a lower percentage of college or higher education (46% versus 58%), but a higher percentage of current smokers (22% versus 10%), heavy alcohol consumption (13% versus 5%), and median waist circumference (98 versus 93 cm). The differences between vegetarian Groups 1–3 are shown in Supplementary Table 4. Vegetarians in Group 3 were younger, more highly educated, and had higher physical activity compared with the other two groups.

The adjusted odds ratios for each outcome are shown in Fig. 1 and Table 4. Group 3 (combined exposure) has the same direction of effect compared to Group 1. However, it shows higher uncertainty and wider CIs in all four outcomes.

**Figure 1:**
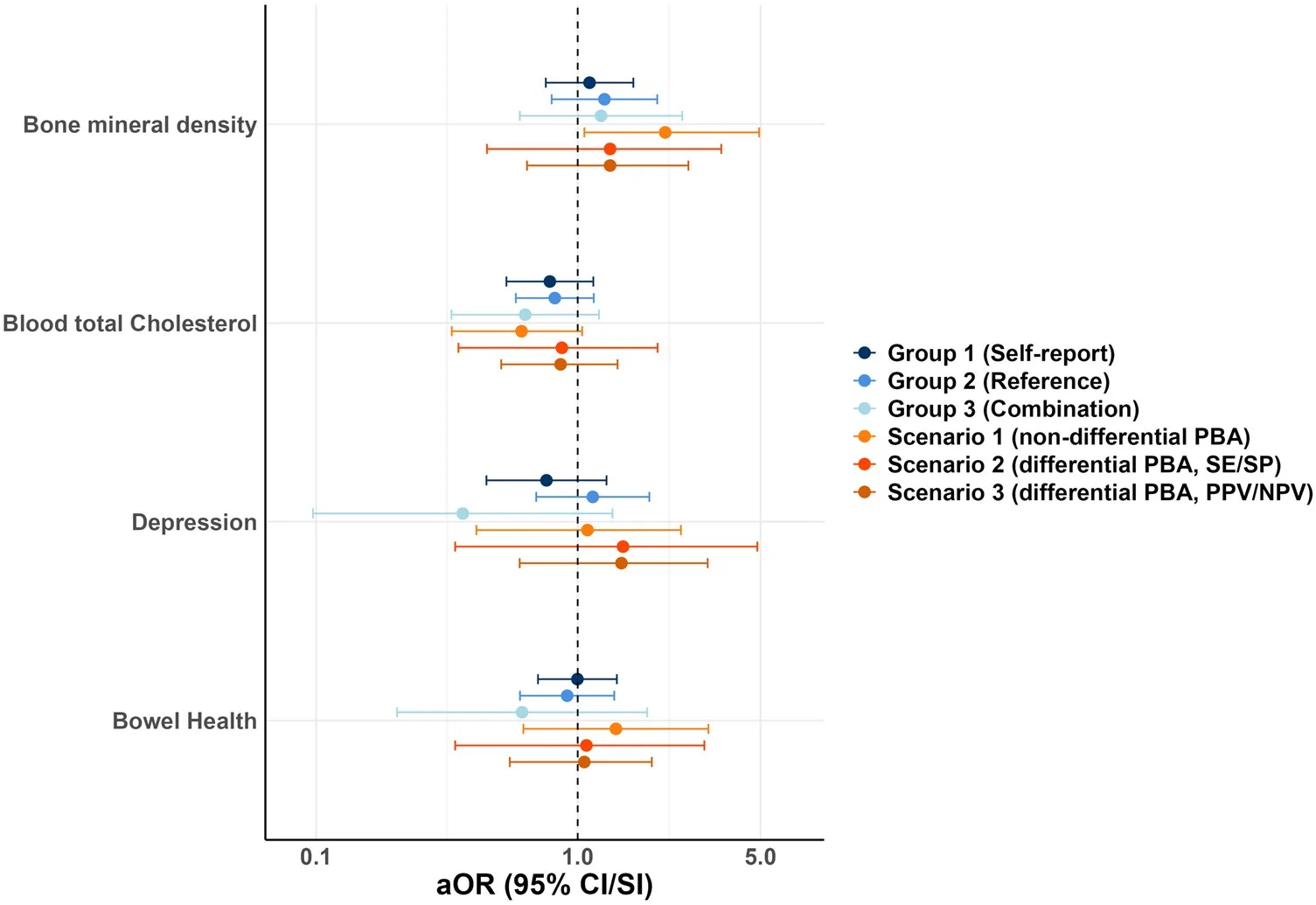
Effect of exposure misclassification (diet type) on adjusted odds ratio (aOR) for four health outcomes (bone mineral density, blood total cholesterol, depression, and bowel health). Group 1 uses self-report as the exposure, Group 2 uses 24-hour dietary recall, Group 3 uses adjusted self-report with 24-hour dietary recall data, Scenario 1 is probabilistic bias analysis with non-differential prior (non-differential PBA), Scenario 2 is probabilistic bias analysis with differential prior distributions for sensitivity and specificity (differential PBA, SE/SP), and Scenario 3 is probabilistic bias analysis with differential prior distributions for positive and negative predictive values (differential PBA, PPV/NPV). SI, simulation interval. Note that the SI values in Scenarios 1 and 2 show the sum of random and systematic error.

**Table 4:** QBA of exposure (diet type) misclassification for four health outcomes [adjusted odds ratio (95% CI)]. Group 1 uses self-report as the exposure, Group 2 uses 24-hour dietary recall, Group 3 uses adjusted self-report with 24-hour dietary recall data, Scenario 1 is probabilistic bias analysis with non-differential prior (non-differential PBA), Scenario 2 is probabilistic bias analysis with differential prior distributions for sensitivity and specificity (differential PBA, SE/SP), and Scenario 3 is probabilistic bias analysis with differential prior distributions for positive and negative predictive value (differential PBA, PPV/NPV). SI, simulation interval. Note that the SI values in Scenarios 1 and 2 show the sum of random and systematic error.

Outcome	Group 1 (self-report)	Group 2 (reference)	Group 3 (combination)	Scenario 1 (non-differential)	Scenario 2 (differential, SE/SP)	Scenario 3 (differential, PPV/NPV)
**Bone mineral density**	1.11 (0.76, 1.63)	1.27 (0.8, 2.01)	1.23 (0.60, 2.51)	2.16 (1.06, 4.94)	1.33 (0.45, 3.54)	1.33 (0.64, 2.65)
**Blood total cholesterol**	0.78 (0.53, 1.15)	0.82 (0.58, 1.15)	0.63 (0.33, 1.21)	0.61 (0.33, 1.04)	0.87 (0.35, 2.02)	0.86 (0.51, 1.42)
**Depression**	0.76 (0.45, 1.29)	1.14 (0.69, 1.88)	0.36 (0.10, 1.36)	1.09 (0.41, 2.48)	1.49 (0.34, 4.86)	1.47 (0.60, 3.14)
**Bowel health**	0.997 (0.70, 1.41)	0.91 (0.60, 1.38)	0.61 (0.20, 1.84)	1.40 (0.62, 3.16)	1.08 (0.34, 3.05)	1.06 (0.55, 1.92)

Figure 2 shows the distribution of sensitivity and specificity values for Scenario 1. The distributions indicate greater uncertainty in sensitivity compared with specificity. In the non-differential misclassification bias analysis (Scenario 1), the adjusted estimates for bone mineral density, total blood cholesterol, and bowel health are shifted away from the null compared with the estimates for exposure Group 1.

**Figure 2:**
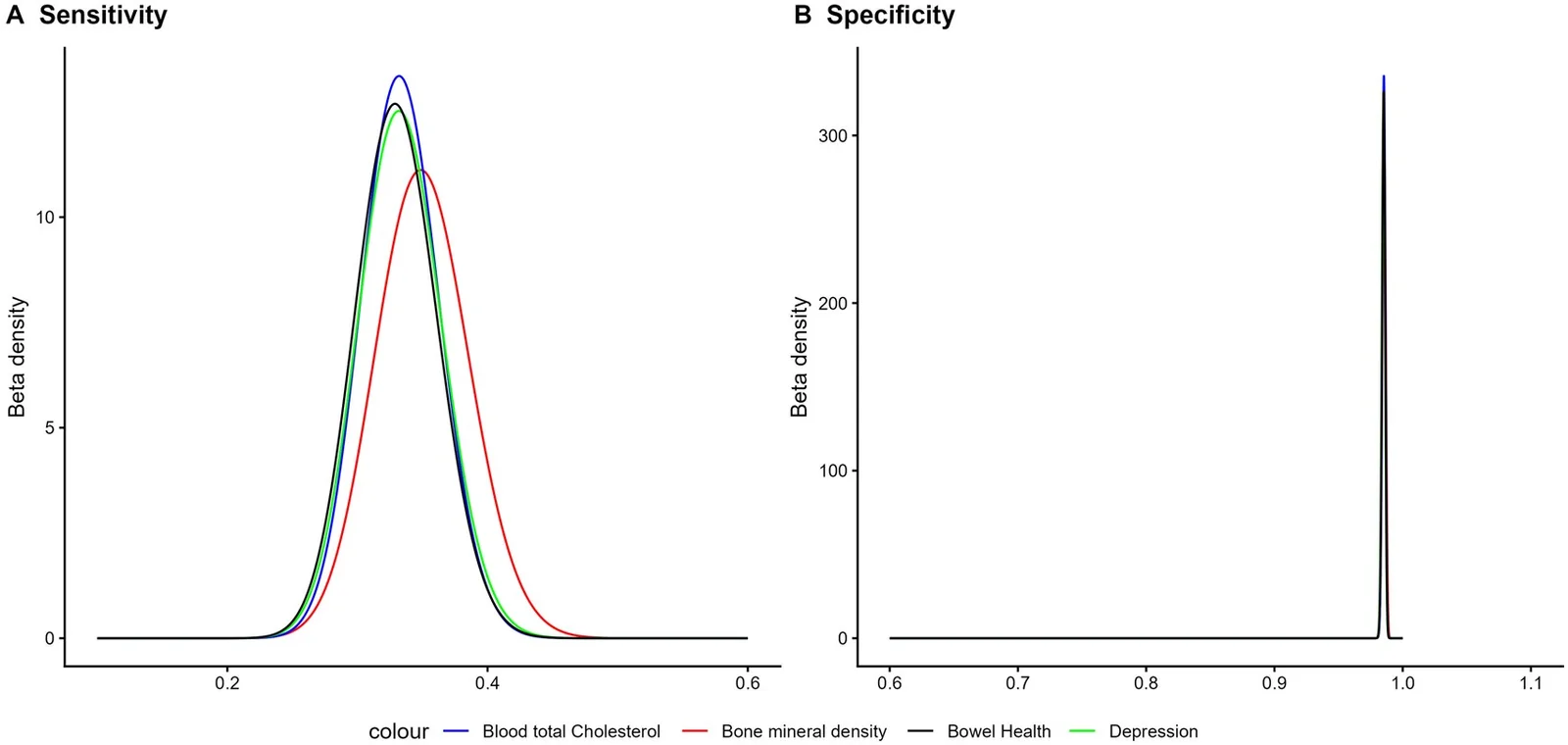
Beta distributions of sensitivity and specificity for each outcome in Scenario 1 (non-differential PBA). The α and β parameters were based on 2 × 2 tables of group 1 (self-report) and group 2 (reference) exposure classification groups.

Scenario 2, differential misclassification analysis, shows a median estimate close to the odds ratios in Group 2 for bone mineral density, blood total cholesterol, and bowel health. The Scenario 3 results are similar to the Scenario 2 results, with a smaller 95% CI. In Scenario 3, the estimates for conventional PBA and PBA with a uniform prior were essentially the same (Supplementary Table 5). The only notable difference was for depression, where the bias-adjusted odds ratio shifted slightly (from 1.41 with conventional PBA to 1.47 with the uniform prior). The latter is closer to the results observed in Scenario 2 (Odds Ratio = 1.49). For depression, the adjusted estimates in Scenarios 1, 2, and 3 are toward and beyond the null.

Assuming a correlation of 0.8 between bias parameters for the exposure did not alter the results, but revealed narrower simulated intervals (Supplementary Table 5).

## Discussion

In this study, we showed the effect of exposure misclassification on evaluating the health effects of a vegetarian diet on four outcomes by using three exposure classification groups and three bias-adjusted scenarios with a probabilistic approach. Using 24h-DRs as the reference reveals low sensitivity and high specificity for self-reported exposure. Although the ideal reference group is not known, it is clear that an absence of meat, poultry, or seafood intake in 2 days of the 24h-DRs does not invalidate a non-vegetarian self-report. The results of the exposure classification groups are different and inconsistent in the amount and direction of effect.

Although one might expect that classification Group 3 (combined) would improve exposure accuracy by providing a more specific measurement (with the same sensitivity), the results for this classification group differ from the bias-adjusted results. The wider confidence interval is likely due to the smaller size of the vegetarian group and not the added uncertainty of the exposure measure [18]. These results showed that a combined classification group provides different results and cannot substitute for QBA. It should be noted, however, that we used 24h-DRs as a self-report-independent reference, without evaluating whether Group 3 might be more appropriate.

Scenario 1 (non-differential PBA) shows an adjusted effect away from the null, with bigger effect sizes and higher uncertainty intervals. Scenario 2 (differential PBA) also mostly shows an adjusted effect away from the null, with the widest uncertainty interval. The wider intervals in Scenarios 1 and 2 could also be due to incorporation of the random error. These scenarios indicate the importance of performing bias analysis. Our study confirms the study of Yland *et al.*, showing that non-differential misclassification of the exposure does not necessarily bias the results toward the null [27].

The median in Scenario 2 is expected to be the same as the adjusted odds ratio of exposure classification in Group 2 because the 24h-DRs were used as the reference for self-reported exposure. However, the weights recommended by the NHANES, along with the covariates included in each model, might explain the observed discrepancies. Additionally, the discrepancy observed in the depression model might be attributed to the low number of vegetarians with the outcome. In all three scenarios, exposure is reclassified in every simulation. However, the weights and covariates remain the same. This would lead to no problem where the exposure is independent of weights and other covariates. However, we think that, in this study, the diet type (exposure) might be associated with the weights and the covariates. In addition, there is a possibility of dependent misclassification because, in exposure classification Group 1, depression and bowel health data were collected via self-reported questionnaires. As a result, the median in Scenario 2, especially for depression and bowel health, is different from the odds ratio in exposure classification Group 2.

Scenario 3 uses PPV and NPV as bias parameters, which are dependent on the exposure prevalence. For our study, this approach is possible because our internal validation is applied to all participants, ensuring that the exposure prevalence is equal between the observed and validation data. However, for other studies in which only external validation is available or the validation data do not match the exposure prevalence of the observed data, relying on sensitivity and specificity is a more reliable approach for bias-adjusting the exposure misclassification. The biggest advantage of this approach is avoiding the negative cells in PBA.

Due to the low number of missing values in the exposure, we assume no significant difference due to missingness. The aim of our study was not to make assumptions about the effect of a vegetarian diet, but to show the importance of exposure misclassification bias analysis in the results of such studies. Studies showing the benefits and harms of vegetarian diets have become popular in recent years and there is an effort to make them credible [3, 7, 28, 29]. However, Fox *et al.* argue that, without measuring the effect of different sources of bias on study results, it is not intuitive to plan where the limited study resources should be spent [30]. In PBA, we used fixed bias parameters to quantify the effect of exposure misclassification on each outcome. In future studies, we aim to study the implications of variation in the bias parameters in different directions, specifically examining the effects of increasing SE/PPV and decreasing SP/NPV on the effect estimates for PBA.

It is important to note that, in this study, we did not evaluate the effect of other biases. Outcome and other covariates were assumed to be measured without error. We also acknowledge that repeated 24h-DRs are not the gold standard for identifying vegetarians and have their limitations [31, 32]. In addition, the vegetarian diet can be chosen for different motivations and has cultural and religious aspects to it, which could affect a person’s self-report of their diet and their health outcomes [7, 33–36]. To adapt to more recent diets and lifestyles, studies should perhaps account for diet as a continuous variable and avoid hard cutoffs where possible. Such approaches allow more flexible diets and different amounts of animal-sourced food, which lead to more informative results.

## Conclusion

To estimate the sensitivity and specificity as well as positive and negative predictive values, dietary assessment instruments such as 24h-DRs can be used, but these only serve as a reference instrument and do not have the properties of a gold standard. Our study showed that categorizing vegetarian and non-vegetarian diets by using self-report, reference method, and as a combination of self-report and reference method led to divergent results. It also showed that the assumption about the non-differential or differential exposure misclassification can affect the results and should, therefore, be meaningfully investigated and justified.

## Ethics approval

NHANES data: Protocol #2005–06, National Center for Health Statistics Ethics Review Board. See https://www.cdc.gov/nchs/nhanes/about/erb.html.

## Supplementary Material

dyag168_Supplementary_Data

## Data Availability

The data are publicly available on the National Health and Nutrition Examination Survey website (https://wwwn.cdc.gov/nchs/nhanes/Default.aspx). Code used in this study is available in the supplementary material.
