# Supplementary material for: Exposure misclassification of self-reported vegetarian diet and its impact on health outcomes
Source: Int J Epidemiol. 2026 Oct 6;55(5):dyag168. doi: 10.1093/ije/dyag168 (PMC13641060; doi:10.1093/ije/dyag168)
Supplement: Supplementary file 1 [file dyag168_supplementary_data.zip › Final_Supplementary_material.pdf]

Exposure misclassification of self-reported vegetarian diet and the impact on health outcomes

Narges Ghoreishi[1,2*], Tobias Kurth[1], Sven Knüppel[3], Matthew P. Fox[4,5]

German Federal Institute for Risk Assessment (BfR), Max-Dohrn-Str. 8-10 10589 Berlin, Germany; narges.ghoreishi@bfr.bund.de

[1] Institute of Public Health, Charité - Universitätsmedizin Berlin, Germany
[2] Department of Exposure, German Federal Institute for Risk Assessment (BfR), Berlin, Germany
[3] Department of Food and Feed Safety in the Food Chain, German Federal Institute for Risk Assessment (BfR), Berlin, Germany
[4] Department of Epidemiology, Boston University School of Public Health, Boston, MA, USA
[5] Department of Global Health, Boston University School of Public Health, Boston, MA, USA

The last two authors contributed equally to the work. Shared last authorship.

1. Section 1 ... 2

1.1. Outcome assessment ... 2

1.1.1. Bone Mineral density ... 2

1.1.2. Total Blood Cholesterol ... 3

1.1.3. Depression ... 3

1.1.4. Bowel health ... 3

1.2. Adjustment variables ... 4

1.3. Missing values ... 5

1.4. Negative bias-adjusted cell counts ... 6

2. Section 2 ... 12

Supplementary figure S1: Flowchart of participant selection from the NHANES data for each outcome ... 12

Supplementary table S1: Exposure, outcome, and bias parameters (sensitivity, specificity, positive predictive value, negative predictive value) for differential and non-differential scenarios stratified by outcome status ... 13

Supplementary table S2: Meat/Poultry/Fish consumption among vegetarians and non-vegetarians ... 14

Supplementary table S3: Summary of missing values among vegetarians ... 14

Supplementary Table S4: Comparison of baseline characteristics of vegetarians in exposure classification groups 1 to 3 with the total sample ... 15

Supplementary table S5: The effect of correlation of bias parameters on the odds ratio [95% SI] of the study estimate, sensitivity/specificity, positive and negative predictive values between the exposed and the unexposed for PBA in scenario 1 (non-differential PBA), in scenario 2 (differential PBA, SE/SP), and in scenario 3 (differential PBA, PPV/NPV) ... 16

3. References ... 17

1. Section 1

1.1. Outcome assessment

We conducted a literature search to identify existing studies that examined self-reported vegetarian diet within National Health and Nutrition Examination Survey (NHANES), yielding three relevant articles. Building on these articles, we investigated the effects of a vegetarian diet on the three outcomes identified in these studies, as well as an additional outcome, blood cholesterol level, which has also been previously associated with a vegetarian diet [1]: 1) bone mineral density, 2) blood total cholesterol, 3) depression, and 4) bowel health. To demonstrate the value of quantitative bias analysis (QBA) to assess the impact of misclassification on the effect of vegetarian diet, we attempted to replicate analyses or the effect of vegetarian diet in publications that used the self-report as exposure and used the same two cycles of the NHANES for bone mineral density, depression, and bowel health. Although a replication of these studies was not feasible for exposure misclassification analysis, we matched, where possible, other elements of data analysis (inclusion and exclusion criteria, data cleaning, confounding variables adjusted for, etc.) from the following studies. Karavasiloglou et al. [2] studied the effect of vegetarian diet on bone mineral density and the possible confounding effect of body mass index. Storz et al. sought to use NHANES data to investigate the effect of vegetarian diet on depression [3] and bowel health [4].

1.1.1. Bone Mineral density

In NHANES bone measurements were obtained using Dual-energy X-ray Absorptiometry (DXA) scans for pelvis, ribs, thoracic and lumbar spine in the mobile examination center. Reasons for exclusion were pregnancy, history of radiographic

contrast material in the past 7 days and weight over 300 pounds or height over 6’5” [5]. Femoral bone mineral density (g/cm^2) data was calculated from these scans, which we categorized those with osteopenia based on the WHO definition [6] of one standard deviation lower than the average value of bone density for women between 20 and 29 years of age for women and the average value of bone density in men between 20 and 29 years of age for men [7, 8].

1.1.2. Total Blood Cholesterol

Despite NHANES examinations taking place twice a day, blood cholesterol levels were collected via a blood sample from participants who attended the mobile examination center in the morning and fasted between 8.5 to 24 hours [5]. Participants were randomly assigned to exams in the morning or in the afternoon. We defined normal total cholesterol levels with blood levels up to 200 mg/dL and high above 200 mg/dL.

1.1.3. Depression

In NHANES, depression status was assessed based on the nine-item Patient Health Questionnaire 9 (PHQ9) depression screening instrument, which was asked by trained interviewers during a private interview in a mobile examination center. PHQ9 evaluates depression symptoms over the past two weeks. Response categories for each item were assigned points: 0 for “not at all”, 1 for “several days”, 2 for “more than half the days”, and 3 for “nearly every day” [5]. We used the same definition of depression as was used by Storz et al. as an overall score of 10 or more [3].

1.1.4. Bowel health

In NHANES, bowel health was evaluated in a mobile examination center via interview data on fecal incontinence and defecation function [5]. Stool inconsistency was assessed using the Bristol Stool Form on a scale of 1-7 (1 being separate hard lumps similar to nuts and 7 being watery consistency, no solid pieces). Storz et al. defined constipation as a score of 1 or 2, and diarrhea as score of 6 or 7. They defined fecal

incontinence as any involuntary loss or accidental leakage of mucus, liquid, and solid stool [4]. We used the same definitions and combined the stool inconsistency and fecal incontinence into a composite binary bowel health outcome so that 0 is when both are negative and 1 is when either one is present.

1.2. Adjustment variables

We modeled bone mineral density (presence or absence of osteopenia) as a function of vegetarian diet (vegetarian, non-vegetarian). We included covariates from the final model of Karavasiloglou et al. [2]: age at study entry (years), race/ethnicity (white, black, mexican American, others), education (high school or lower, college or higher), menopausal status (premenopause, postmenopause, male), smoking (non.smoker, former smoker, non-smoker), marital status (married/living with partner, divorced/separated, widowed/never married), physical activity (no physical activity, moderate to vigorous), alcohol consumption (non-drinker, binge drinker, moderate drinker, heavy drinker), vitamin D (user, non-user), body mass index (BMI, cm/kg^2), and waist circumference (cm). In contrast to the cited model, we excluded sex (male, female) and calcium supplement (user, non-user) for collinearity purposes, as these categories were mirrored by menopausal status and vitamin D.

We modeled blood cholesterol (normal, high) as a function of vegetarian diet (vegetarian, non-vegetarian). We adjusted the model for age (years), sex (male, female), ethnicity/race (white, black, mexican American, others), education level (high school or lower, college or higher), smoking (non.smoker, former smoker, non-smoker) and alcohol status (non-drinker, binge drinker, moderate drinker, heavy drinker), physical activity (no physical activity, moderate to vigorous), and BMI (cm/kg^2).

We modeled depression (depressed, not-depressed) as a function of vegetarian diet (vegetarian, non-vegetarian) and adjusted for age at study entry (years), sex (male,

female), ethnicity/race (white, black, mexican American, others), annual household income (dollars), marital status (married/living with partner, divorced/separated, widowed/never married), smoking (non.smoker, former smoker, non-smoker), education (high school or lower, college or higher), BMI (cm/kg^2), and C-reactive protein (mg/dL) as was done by Storz et al. [3].

We modeled bowel health (not-healthy, healthy) as a function of vegetarian diet (vegetarian, non-vegetarian). We adjusted for age at study entry (years), sex (male, female), ethnicity/race (white, black, Mexican American, others), annual household income (dollars), marital status (married/living with partner, divorced/separated, widowed/never married), and BMI (kg/m^2) [4].

1.3. Missing values

Participants who only answered one of the 24-hour dietary recalls were assessed based on that day. Our handling of missing values is aligned with the referenced study for other variables. For bone mineral density, Karavasiloglou labeled missing variables as an extra category with an “unknown” label [2]. We took the same approach for blood total cholesterol. For bowel health and depression, the two studies of Storz et al. performed a complete case analysis [3, 4].

For the purposes of comparing the exposure classification groups 1-3, we have excluded the participants who did not have dietary recall. However, when the aim of the study is to measure the effect of exposure on outcome, these participants could be included. Note that here we applied complete case analysis of exposure and outcome measures, which might not be the ideal method to deal with missing data.

1.4. Negative bias-adjusted cell counts

In scenario 2, when applying probabilistic bias analysis (PBA), we obtained negative bias-corrected cell counts in simulation repetitions, which indicates an inconsistency between the observed data and the assumed bias parameters. Scenario 1 resulted in there were no negative cell counts. Negative bias-adjusted cell counts are implausible, signalling a problem with the assumed parameter. Hence, to ensure a meaningful (defined and positive) bias-adjusted odds ratio, two sets of inequalities provides boundaries for sensitivity and specificity among diseased ($d = 1$) and undiseased ($d = 0$) under differential misclassification that avoid negative bias-adjusted cell counts derived from the formulas given in **supplementary table a and b** are given [9,10]

$1 - Pr(E^* = 1 \mid D = d) \leq SP_D \leq 1$ and $Pr(E^* = 1 \mid D = d) \leq SE_D \leq 1$

or

$0 \leq SP_D \leq 1 - Pr(E^* = 1 \mid D = d)$ and $0 \leq SE_D \leq Pr(E^* = 1 \mid D = d)$

$Pr(E^* = 1 \mid D = d)$ denotes the observed exposure probability within level d of the diseased group ($d = 1$) and $d = 0$ for undiseased group.

The observed exposure probability for the diseased group ($d = 1$) is $Pr(E^* = 1 \mid D = 1) = \frac{a}{a+b}$, and for the undiseased group ($d = 0$) it is $Pr(E^* = 1 \mid D = 0) = \frac{c}{c+d}$. Consequently, it is $1 - Pr(E^* = 1 \mid D = 1) = \frac{b}{a+b}$ and $1 - Pr(E^* = 1 \mid D = 0) = \frac{d}{c+d}$. The calculated threshold values were shown in **supplementary table c**.

Supplementary table a: Equations for calculating bias-adjusted cell counts in

	Bias-adjusted cell counts	
	**Exposed (E = 1)**	**Unexposed (E = 0)**
**Diseased (D = 1)**	$A = \frac{a - (a + b)(1 - SP_{D=1})}{SE_{D=1} + SP_{D=1} - 1}$	$B = a + b - \frac{a - (a + b)(1 - SP_{D=1})}{SE_{D=1} + SP_{D=1} - 1}$
**Undiseased (D = 0)**	$C = \frac{c - (c + d)(1 - SP_{D=0})}{SE_{D=0} + SP_{D=0} - 1}$	$D = c + d - \frac{c - (c + d)(1 - SP_{D=0})}{SE_{D=0} + SP_{D=0} - 1}$

scenario 1 and 2, taken from Fox et al. [10]

a, b, c, d: observed cell counts of the 2x2 contingency tables for exposure (E=1, E=0) and outcome (D=1, D=0) pairs as given in Table 3 of the article.

$SE_{D=1}$, $SE_{D=0}$, $SP_{D=1}$, $SP_{D=0}$: sensitivity (SE) and specificity (SP) for the diseased (D=1) and the undiseased (D=0), respectively.

Supplementary table b: Equations for calculating bias-adjusted cell counts in scenario 3, taken from Fox et al. [10]

	Bias-adjusted cell counts	
	**Exposed (E = 1)**	**Unexposed (E = 0)**
**Diseased (D = 1)**	$A = a(PPV_{D1}) + b(1 - NPV_{D1})$	$B = D_{1\,Total} - A$
**Undiseased (D = 0)**	$C = c(PPV_{D0}) + d(1 - NPV_{D0})$	$D = D_{0\,Total} - C$

a, b, c, d: observed cell counts of the 2x2 contingency tables for exposure (E=1, E=0) and outcome (D=1, D=0) pairs as given in Table 3 of the article.

$PPV_{D=1}$, $PPV_{D=0}$, $NPV_{D=1}$, $NPV_{D=0}$: positive predictive value (PPV) and negative predictive value (NPV) for the diseased (D=1) and the undiseased (D=0), respectively.

If $SE_{D=1} = \frac{a}{a+b}$, $SP_{D=1} = \frac{b}{a+b}$, $SE_{D=0} = \frac{c}{c+d}$ or $SP_{D=0} = \frac{d}{c+d}$, then one cell count in the bias-adjusted 2x2 table becomes zero.

If (1) $SE_{D=1} = \frac{a}{a+b}$ and $SP_{D=1} = \frac{b}{a+b}$, or (2) $SE_{D=0} = \frac{c}{c+d}$ and $SP_{D=0} = \frac{d}{c+d}$, the bias-adjusted odds ratio is undefined because one denominator in the cell-count calculation is zero.

Supplementary table c: Threshold values based on observed cell counts (a, b, c, and d) from observed self-reported dietary types (exposure) and four health outcomes based on **Supplementary table S1**.

	$1 - Pr(E^* = 1 \mid D = 1) = \dfrac{b}{a+b}$	$Pr(E^* = 1 \mid D = 1) = \dfrac{a}{a+b}$	$1 - Pr(E^* = 1 \mid D = 0) = \dfrac{d}{c+d}$	$Pr(E^* = 1 \mid D = 0) = \dfrac{c}{c+d}$
**Bone mineral density**	$\frac{2277}{62+2277} = 0.9735$	$\frac{62}{62+2277} = 0.0265$	$\frac{5632}{115+5632} = 0.9800$	$\frac{115}{115+5634} = 0.0200$
**Blood total cholesterol**	$\frac{4492}{94+4492} = 0.9795$	$\frac{94}{94+4492} = 0.0205$	$\frac{5760}{5760+140} = 0.9763$	$\frac{140}{5760+140} = 0.0237$
**Depression**	$\frac{911}{22+911} = 0.9764$	$\frac{22}{22+911} = 0.0236$	$\frac{8577}{194+8577} = 0.9779$	$\frac{194}{194+8577} = 0.0221$
**Bowel health**	$\frac{2262}{56+2262} = 0.9758$	$\frac{56}{56+2262} = 0.0242$	$\frac{7522}{164+7522} = 0.9787$	$\frac{164}{164+7522} = 0.0213$

Assuming beta distribution, we calculated the probability for negative bias-adjusted cell counts for cells A and B as well as C and D for each of the four outcomes.

$$\boldsymbol{SE_{D=1}^{Beta} \sim Beta\left(\alpha_{SE_{D=1}}, \beta_{SE_{D=1}}\right)}$$

$$\boldsymbol{SP_{D=1}^{Beta} \sim Beta\left(\alpha_{SP_{D=1}}, \beta_{SP_{D=1}}\right)}$$

$$Prob(negative\ bias - adjusted\ cell\ counts\ of\ cells\ A\ and\ B) =$$

$$1 - Prob(zero\ and\ nonnegative\ bias - adjusted\ cell\ counts\ of\ cells\ A\ and\ B) =$$

$$1 - \left[\left(1 - P\left(SE_{D=1}^{Beta} \le \frac{a}{a+b}\right)\right) \cdot \left(1 - P\left(SP_{D=1}^{Beta} \le \frac{b}{a+b}\right)\right) + P\left(SE_{D=1}^{Beta} \le \frac{a}{a+b}\right) \cdot P\left(SP_{D=1}^{Beta} \le \frac{b}{a+b}\right)\right]$$

Analogously, the probability for negative bias-adjusted cell counts for cells C and D can be calculated.

Note that for any continuous probability distribution X, as with beta distribution, the probability of it for any specific value x is zero. Formally, this is expressed as $P(X = x) = 0$. Accordingly, for example $P\left(SE_{D=1}^{Beta} = \frac{a}{a+b}\right) = 0$ and $P\left(SP_{D=1}^{Beta} = \frac{b}{a+b}\right) = 0$. Thus, in the case of the beta distribution, $P\left(SE_{D=1}^{Beta} < \frac{a}{a+b}\right) = P\left(SE_{D=1}^{Beta} \le \frac{a}{a+b}\right)$ and $P\left(SP_{D=1}^{Beta} < \frac{b}{a+b}\right) = P\left(SP_{D=1}^{Beta} \le \frac{b}{a+b}\right)$ applies here. Hence, the probability that either

of the two specific situations mentioned is zero when applying the beta distribution to obtain a bias-adjusted cell count of zero or negative.

We estimated the probability of negative bias-adjusted cell counts using observed self-reported dietary exposure and observed outcome data (a, b, c, d) and internal validation data from 24-hour dietary recalls, and compared this to the counts of PBA repetitions yielding negative bias-adjusted cells. Results showed strong agreement between the estimated probability and the observed number of negative repetitions (**supplementary Table d**). This indicated that the simulation of sensitivity and specificity was reasonable.

Selection of bias parameter (α and β) for beta distribution $\boldsymbol{Beta(\alpha, \beta)}$

For the four outcomes, by using the boundaries derived above, we found factors for α and β using the beta distribution, where the sum of probability of negative bias-adjusted cell counts of A and B as well as C and D were below the threshold $\varepsilon = 0.001\%$. To achieve this, we examined a grid of integer values for $k_{SE_{D=1}}$, $k_{SP_{D=1}}$,$k_{SE_{D=0}}$ and $k_{SP_{D=0}}$, ranging each from 1 to 100, considering all combinations where the probability of negative bias-adjusted cell counts were below 0.001%. We chose that combinations which had the lowest sum of $k_{SE_{D=1}}$, $k_{SP_{D=1}}$,$k_{SE_{D=0}}$ and $k_{SP_{D=0}}$. The selected factors which were chosen are shown in **Supplementary table e**. As sensitivity analysis in scenario 2, we applied PBA multiplying the α and β parameter of the beta distribution by 10 with the exception of depression, where the parameters were multiplied by 100. As sensitivity analysis for scenario 3, we compared the results of uniform prior with conventional approach. The detailed description and comparison of these two approaches are explained by Zhang et. al [11].

Supplementary table d: Probability of negative bias-adjusted cell counts based on observed self-reported dietary types (exposure) and outcome data (a, b, c, and d) and internal validation data based on 24-hour dietary recalls and count of negative bias-adjusted cell counts in Scenario 2.

	**Bias-adjusted cells A and B**	**Bias-adjusted cells C and D**	**Scenario 2 without changing α and β**
	SE_{D1}^{Beta}<br>SP_{D1}^{Beta}<br>***Prob(negative bias-adjusted cell counts)***<br><br>Expected number of repetitions with negative bias-adjusted cell counts (Rounded down to the nearest integer) out of 100.000 repetitions	SE_{D0}^{Beta}<br>SP_{D0}^{Beta}<br>***Prob(negative bias-adjusted cell counts)***<br><br>Expected number of repetitions with negative bias-adjusted cell counts (Rounded down to the nearest integer) out of 100.000 repetitions	Proportion of PBA simulation repetitions with negative cell counts - differential exposure misclassification (100,000 simulation repetitions)
**Bone mineral density**	$Beta(21, 43)$<br>$Beta(2234, 41)$<br>**0.00326 = 0.326%**<br><br>326 out of 100.000	$Beta(41, 72)$<br>$Beta(5560, 74)$<br>$\mathbf{3.7 \cdot 10^{-5} = 0.0037}\%$<br><br>3 out of 100.000	334 / 100,000 = 0.334%
**Blood total cholesterol**	$Beta(24, 77)$<br>$Beta(4415, 70)$<br>**0.00715 = 0.715%**<br><br>715 out of 100.000	$Beta(59, 89)$<br>$Beta(5671, 81)$<br>$\mathbf{8.0 \cdot 10^{-8} = 0.000008}\%$<br><br>0 out of 100.000	726 / 100,000 = 0.726%
**Depression**	$Beta(4, 25)$<br>$Beta((886, 18)$<br>**0.20801 = 20.801%**<br><br>20801 out of 100.000	$Beta(69, 121)$<br>$Beta(8456, 125)$<br>$\mathbf{8.4 \cdot 10^{-10} = 8.4 \cdot 10^{-8}}\%$<br><br>0 out of 100.000	20929 / 100,000 = 20.9%
**Bowel health**	$Beta(12, 43)$<br>$Beta(2219, 44)$<br>**0.05945 = 5.945%**<br><br>5945 out of 100.000	$Beta(62, 107)$<br>$Beta(7415, 102)$<br>$\mathbf{2.6 \cdot 10^{-7} = 0.000026}\%$<br><br>0 out of 100.000	5785 / 100,000 = 5.8%

The sensitivity analysis of approaches to avoid negative cells counts for scenario 2 are shown in **Supplementary table e**. The resulting bias-adjusted odds ratios did not change essentially but the simulated intervals were smaller. Since no negative bias-adjusted cell counts were observed in scenario 1 and 3, no adjustment of bias parameters were done in these scenarios.

Supplementary table e: Comparison of different chosen factors for the bias parameters and their corresponding negative cells in the bias-adjusted exposure for the PBA in scenario 2 (differential, PBA SE/SP)

Outcome	Factor	Odds ratio [95% CI]	Number of negative cells*
**Bone mineral density**	$k\_se_{D=1} = 1$, $k\_sp_{D=1} = 1$<br>$k\_se_{D=0} = 1$, $k\_sp_{D=0} = 1$	1.33 [0.29, 4.27]	334
	$k\_se_{D=1} = 10$, $k\_sp_{D=1} = 10$<br>$k\_seD_{=0} = 10$, $k\_sp_{D=0} = 10$	1.32 [0.59, 2.78]	0
	ε = 0.001%<br>$k\_se_{D=1} = 1$, $k\_sp_{D=1} = 3$<br>$k\_se_{D=0} = 1$, $k\_sp_{D=0} = 2$	1.33 [0.45, 3.54]	0
**Blood total cholesterol**	$k\_se_{D=1} = 1$, $k\_sp_{D=1} = 1$<br>$k\_se_{D=0} = 1$, $k\_sp_{D=0} = 1$	0.86 [0.19, 2.25]	726
	$k\_se_{D=1} = 10$, $k\_sp_{D=1} = 10$<br>$k\_se_{D=0} = 10$, $k\_sp_{D=0} = 10$	0.86 [0.24,1.68]	0
	ε = 0.001%<br>$k\_se_{D=1} = 1$, $k\_sp_{D=1} = 4$<br>$k\_se_{D=0} = 1$, $k\_sp_{D=0} = 1$	0.87 [0.35, 2.02]	0
**Depression**	$k\_se_{D=1} = 1$, $k\_sp_{D=1} = 1$<br>$k\_se_{D=0} = 1$, $k\_sp_{D=0} = 1$	2.14 [0.06, 17.59]	20929
	$k\_se_{D=1} = 100$, $k\_sp_{D=1} = 100$<br>$k\_se_{D=0} = 100$, $k\_sp_{D=0} = 100$	1.42 [0.51,3.16]	0
	ε = 0.001%<br>$k\_se_{D=1} = 3$, $k\_sp_{D=1} = 33$<br>$k\_se_{D=0} = 1$, $k\_sp_{D=0} = 1$	1.49 [0.34, 4.86]	0
**Bowel health**	$k\_se_{D=1} = 1$, $k\_sp_{D=1} = 1$<br>$k\_se_{D=0} = 1$, $k\_sp_{D=0} = 1$	1.14 [0.09, 3.85]	5785
	$k\_se_{D=1} = 10$, $k\_sp_{D=1} = 10$<br>$k\_se_{D=0} = 10$, $k\_sp_{D=0} = 10$	1.06 [0.43, 2.12]	0
	ε = 0.001%<br>$k\_se_{D=1} = 1$, $k\_sp_{D=1} = 8$<br>$k\_se_{D=0} = 1$, $k\_sp_{D=0} = 1$	1.09 [0.35, 3.07]	1

* Number of repetitions with negative bias-adjusted cell counts out of 100000 simulation repetitions

2. Section 2

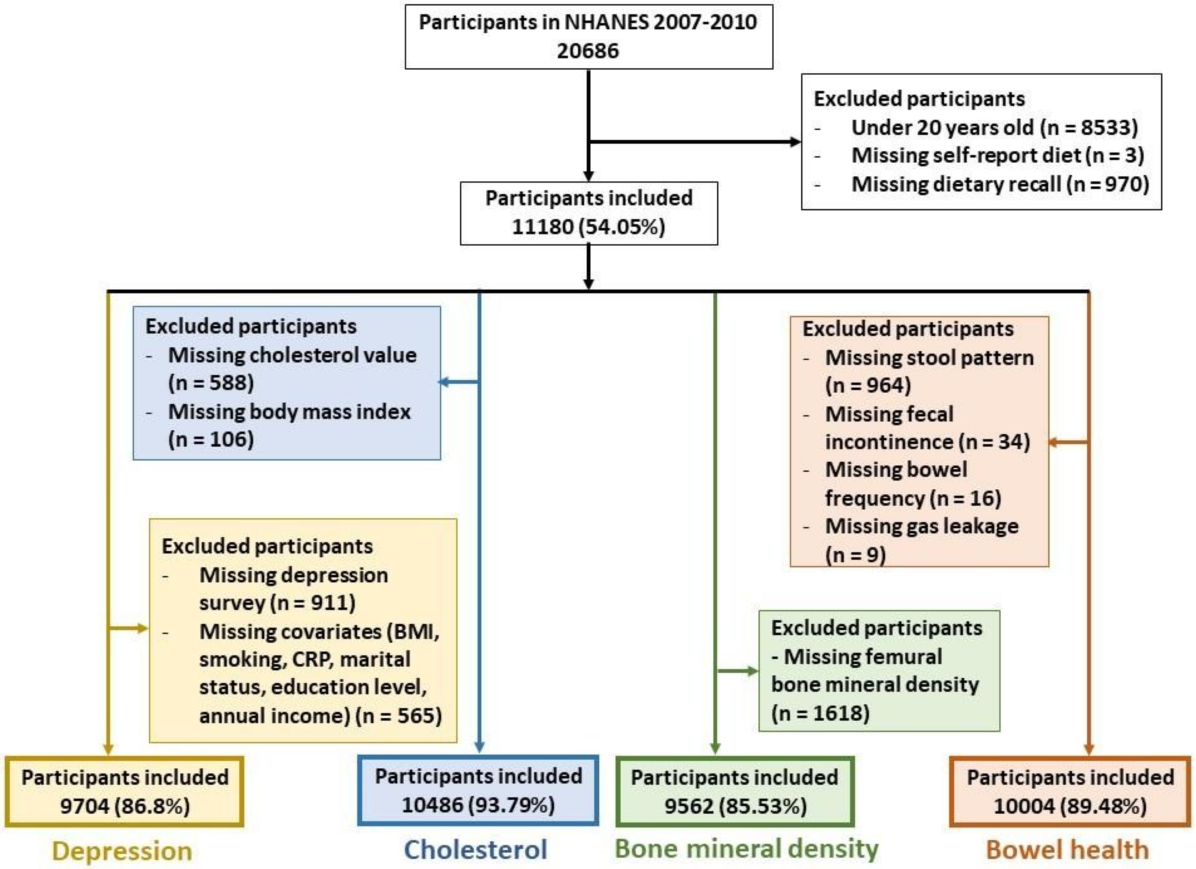

Supplementary Figure S1: Flowchart of participant selection from the National Health and Nutrition Examination Survey data for each outcome.

Supplementary table S1: Exposure, outcome, and bias parameters (sensitivity, specificity, positive predictive value, negative predictive value) for differential and non-differential scenarios stratified by outcome status

Outcome	Status<br>d	a<br>α of SE<br>α of PPV	b<br>β of SP<br>β of PPV	c<br>β of SE<br>β of NPV	d<br>α of SP<br>α of NPV	SE [95% CI]	SP [95% CI]	PPV [95%]	NPV [95% CI]
**Bone mineral density**	Non-differential	62	115	115	7794	0.35 [0.28, 0.43]	0.99 [0.98, 0.99]		
	1	21	41	43	2234	0.33 [0.22,0.46]	0.98 [0.98,0.99]	0.34 [0.22, 0.47]	0.98 [0.97, 0.99]
	0	41	74	72	5560	0.36 [0.27,0.46]	0.99 [0.98,0.99]	0.36 [0.27, 0.45]	0.99 [0.98, 0.99]
**Blood total cholesterol**	Non-differential	83	151	166	10086	0.33 [0.28, 0.40]	0.99 [0.98, 0.99]		
	1	24	70	77	4415	0.24 [0.16,0.33]	0.98 [0.98,0.99]	0.26 [0.17, 0.36]	0.98 [0.98, 0.99]
	0	59	81	89	5671	0.40 [0.32,0.48]	0.99 [0.98,0.99]	0.42 [0.34, 0.51]	0.99 [0.98, 0.99]
**Depression**	Non-differential	73	143	146	9342	0.33 [0.27, 0.40]	0.98 [0.98, 0.99]		
	1	4	18	25	886	0.14 [0.04,0.32]	0.98 [0.97,0.99]	0.18 [0.05, 0.40]	0.97 [0.96, 0.98]
	0	69	125	121	8456	0.36 [0.29,0.44]	0.99 [0.98,0.99]	0.36 [0.29, 0.43]	0.99 [0.98, 0.99]
**Bowel health**	Non-differential	74	146	150	9634	0.33 [0.27, 0.40]	0.99 [0.98, 0.99]		
	1	12	44	43	2219	0.22 [0.12,0.35]	0.98 [0.97,0.99]	0.21 [0.12, 0.34]	0.98 [0.97, 0.99]
	0	62	102	107	7415	0.37 [0.29,0.44]	0.99 [0.98,0.99]	0.38 [0.30, 0.46]	0.99 [0.98, 0.99]

a: Number of self-reported vegetarians with no meat/poultry/seafood consumption in the 24-hour dietary recalls
b: Number of self-reported vegetarians with meat/poultry/seafood consumption in the 24-hour dietary recalls
c: Number of self-reported non-vegetarians with no meat/poultry/seafood consumption in the 24-hour dietary recalls
d: Number of self-reported non-vegetarians with meat/poultry/seafood consumption in the 24-hour dietary recalls
SE: Sensitivity, SP: Specificity
d: d=1 for diseased, d=0 for undiseased

Supplementary table S2: Meat/Poultry/Fish consumption among vegetarians and non-vegetarians

	vegetarian based on self-report and 24h-DR*	vegetarian based on self-report but not 24h-DR*	Non-vegetarians
**Meat/poultry/fish consumption on day 1 and day 2**	0	86	8620
**Meat/poultry/fish consumption only on day 1**	0	52	1742
**Meat/poultry/fish consumption only on day 2**	0	26	390
**Meat/poultry/fish consumption neither on day 1 nor day 2**	84	0	180

*24 hour dietary recall

Supplementary table S3: Summary of missing values among vegetarians

	vegetarian based on self-report	Total
**Missing recall data on only dietary recall day 1**	0	0
**Missing recall data on only dietary recall day 2**	33	1462
**Data on both days are missing**	25	970

Supplementary Table S4: Comparison of baseline characteristics of vegetarians in exposure classification groups 1 to 3 with the total sample

	Group 1	Group 2	Group 3	Total
**Characteristic**	**N = 248[1]**	**N = 264[1]**	**N = 84[1]**	**N = 11,180[1]**
**Age**	51 (35, 64)	50 (35, 67)	40 (31, 55)	49 (35, 64)
**Sex**				
male	95 (38%)	97 (37%)	34 (40%)	5,450 (49%)
female	153 (62%)	167 (63%)	50 (60%)	5,730 (51%)
**Ethnicity**				
Mexican Am	38 (15%)	44 (17%)	7 (8.3%)	1,991 (18%)
NH Black	34 (14%)	25 (9.5%)	8 (9.5%)	2,161 (19%)
NH White	100 (40%)	132 (50%)	40 (48%)	5,333 (48%)
Other	76 (31%)	63 (24%)	29 (35%)	1,695 (15%)
**Education level**				
college or higher	144 (58%)	137 (52%)	65 (77%)	5,208 (47%)
High school or lower	104 (42%)	127 (48%)	19 (23%)	5,955 (53%)
**Marital status**				
divorced/separated	40 (16%)	39 (15%)	11 (13%)	1,615 (14%)
married/living with partner	143 (58%)	142 (54%)	49 (58%)	6,679 (60%)
widowed/never married	65 (26%)	83 (31%)	24 (29%)	2,880 (26%)
Annual household income	8 (5, 13)	8 (5, 14)	9 (6, 14)	7 (5, 14)
**Smoking**				
current smoker	24 (9.7%)	45 (17%)	8 (9.5%)	2,443 (22%)
former smoker	60 (24%)	55 (21%)	18 (21%)	2,778 (25%)
non-smoker	164 (66%)	164 (62%)	58 (69%)	5,957 (53%)
**Alcohol**				
binge drinker	48 (19%)	40 (15%)	19 (23%)	2,230 (20%)
heavy drinker	13 (5.2%)	22 (8.3%)	4 (4.8%)	1,412 (13%)
moderate drinker	59 (24%)	68 (26%)	25 (30%)	3,243 (29%)
non drinker	48 (19%)	44 (17%)	9 (11%)	2,046 (18%)
Unknown	80 (32%)	90 (34%)	27 (32%)	2,249 (20%)
**Menopause**				
postmenopausal	72 (29%)	68 (26%)	14 (17%)	2,705 (24%)
premenopausal	69 (28%)	78 (30%)	29 (35%)	2,540 (23%)
Unknown	12 (4.8%)	21 (8.0%)	7 (8.3%)	485 (4.3%)
**Body mass index**	25.9 (23.1, 30.0)	25.9 (22.9, 30.4)	25.2 (22.3, 29.6)	28 (24, 32)
**Waist circumference, cm**	93 (83, 101)	93 (84, 103)	91 (81, 99)	98 (88, 109)
**Physical activity**				
moderate to vigorous	120 (48%)	122 (46%)	53 (63%)	4,941 (44%)
no	128 (52%)	142 (54%)	31 (37%)	6,239 (56%)
**C reactive protein**	0.14 (0.06, 0.43)	0.17 (0.07, 0.41)	0.15 (0.06, 0.42)	0.20 (0.08, 0.46)
**Serum total 25(OH)D, nmol/L**				
non user	118 (48%)	123 (47%)	34 (40%)	5,914 (53%)
Unknown	36 (15%)	41 (16%)	12 (14%)	1,485 (13%)
user	94 (38%)	100 (38%)	38 (45%)	3,780 (34%)

[1] Median (Q1, Q3); n (%)

Supplementary table S5: The effect of correlation of bias parameters on the odds ratio [95% SI] of the study estimate, sensitivity/specificity, positive and negative predictive values between the exposed and the unexposed for PBA in scenario 1 (non-differential PBA), in scenario 2 (differential PBA, SE/SP), and in scenario 3 (differential PBA, PPV/NPV)

	**Odds ratio [95% SI]**							
**Correlation**	**Rho = 0**	**Rho = 0.8**	**Rho = 0**	**Rho = 0.8**	**Rho = 0**	**Rho = 0.8**	**Rho = 0**	**Rho = 0.8**
**Scenario**	**Scenario 1 (non-differential)**	**Scenario 1 (non-differential)**	**Scenario 2 (differential)**	**Scenario 2 (differential)**	**Scenario 3 (conventional)**	**Scenario 3 (conventional)**	**Scenario 3 (uniform)**	**Scenario 3 (uniform)**
**Bias parameter**	**SE/SP**	**SE/SP**	**SE/SP**	**SE/SP**	**PPV/NPV**	**PPV/NPV**	**PPV/NPV**	**PPV/NPV**
**Bone mineral density**	2.14 [0.87, 6.31]	2.15 [1.02, 5.32]	1.33 [0.45, 3.54]	1.34 [0.56, 2.86]	1.32 [0.63, 2.64]	1.32 [0.66, 2.50]	1.33 [0.64, 2.65]	1.33 [0.68, 2.52]
**Blood total cholesterol**	0.61 [0.25, 1.28]	0.61 [0.30, 1.08]	0.87 [0.35, 2.02]	0.87 [0.44, 1.58]	0.86 [0.50, 1.42]	0.86 [0.53, 1.35]	0.86 [0.51, 1.42]	0.86 [0.53, 1.36]
**Depression**	1.09 [0.34, 2.93]	1.09 [0.39, 2.56]	1.49 [0.34, 4.86]	1.49 [0.43, 3.85]	1.41 [0.55, 3.06]	1.41 [0.59, 2.93]	1.47 [0.60, 3.14]	1.47 [0.63, 3.01]
**Bowel health**	1.42 [0.63, 3.19]	1.42 [0.75, 2.72]	1.09 [0.35, 3.07]	1.09 [0.44, 2.37]	1.04 [0.53, 1.90]	1.04 [0.56, 1.81]	1.06 [0.55, 1.92]	1.06 [0.58, 1.84]

3. References

1. Oussalah A, Levy J, Berthezène C et al. Health outcomes associated with vegetarian diets: An umbrella review of systematic reviews and meta-analyses. Clinical Nutrition 2020;39(11):3283–307.

2. Karavasiloglou N, Selinger E, Gojda J et al. Differences in bone mineral density between adult vegetarians and nonvegetarians become marginal when accounting for differences in anthropometric factors. The Journal of Nutrition 2020;150(5):1266–71.

3. Storz MA, Ronco AL. Adherence to a vegetarian diet is not associated with depression: Results from the national health and nutrition examination surveys. Psychiatry Investigation 2023;20(4):315.

4. Storz MA, Rizzo G, Müller A et al. Bowel health in US vegetarians: A 4-year data report from the national health and nutrition examination survey (NHANES). Nutrients 2022;14(3):681.

5. Centers for Disease Control and Prevention (CDC). National Center for Health Statistics (NCHS). National Health and Nutrition Examination Survey Data. Hyattsville, MD: U.s. Department of Health and Human Services, Centers for Disease Control and Prevention. at: https://www.cdc.gov/nchs/nhanes/index.htm., n.d.

6. Organization WH et al. Assessment of Fracture Risk and Its Application to Screening for Postmenopausal Osteoporosis: Report of a WHO Study Group [Meeting Held in Rome from 22 to 25 June 1992]. n.p.: World Health Organization, 1994.

7. Looker AC, Orwoll ES, Johnston JR CC et al. Prevalence of low femoral bone density in older US adults from NHANES III. Journal of Bone and Mineral Research 1997;12(11):1761–8.

8. Looker AC, Borrud LG, Hughes JP et al. Lumbar spine and proximal femur bone mineral density, bone mineral content, and bone area: United states, 2005-2008. Vital and Health Statistics Series 11, Data from the National Health Survey 2012;(251):1–132.

9. MacLehose RF, Gustafson P. Is probabilistic bias analysis approximately bayesian? Epidemiology 2012;23(1):151–8.

10. Fox MP, MacLehose RF, Lash TL. Applying Quantitative Bias Analysis to Epidemiologic Data. n.p.: Springer, 2021.

11. Zhang Q, MacLehose RF, Collin LJ et al. Parameterization of beta distributions for bias parameters of binary exposure misclassification in probabilistic bias analysis. Epidemiology 2025;36(2):237–44.
